# Ephrin type-A receptor 2 regulates sensitivity to paclitaxel in nasopharyngeal carcinoma via the phosphoinositide 3-kinase/Akt signalling pathway

**DOI:** 10.3892/mmr.2014.2799

**Published:** 2014-10-29

**Authors:** YUNYUN WANG, YONG LIU, GUO LI, ZHONGWU SU, SHULING REN, PINGQING TAN, XIN ZHANG, YUANZHENG QIU, YONGQUAN TIAN

**Affiliations:** 1 Department of Otolaryngology Head and Neck Surgery, Xiangya Hospital, Central South University, Changsha, Hunan 410008, P.R. China; 2 Otolaryngology Major Disease Research Key Laboratory of Hunan Province, Changsha, Hunan 410008, P.R. China

**Keywords:** nasopharyngeal carcinoma, ephrin type-A receptor 2, drug resistance, paclitaxel, phosphoinositide 3-kinase/Akt

## Abstract

Ephrin type-A receptor 2 (EphA2) is a receptor tyrosine kinase that is associated with cancer cell metastasis. There has been little investigation into its impact on the regulation of sensitivity to paclitaxel in nasopharyngeal carcinoma (NPC). In the present study, upregulation of EphA2 expression enhanced the survival of NPC 5-8F cells, compared with control cells exposed to the same concentrations of paclitaxel. Flow cytometry and western blot analysis demonstrated that over-expression of EphA2 decreased NPC cancer cell sensitivity to paclitaxel by regulating paclitaxel-mediated cell cycle progression but not apoptosis *in vitro*. This was accompanied by alterations in the expression of cyclin-dependent kinase inhibitors, p21 and p27, and of inactive phosphorylated-retinoblastoma protein. Furthermore, paclitaxel stimulation and EphA2 over-expression resulted in activation of the phosphoinositide 3-kinase (PI3K)/Akt signalling pathway in NPC cells. Inhibition of the PI3K/Akt signalling pathway restored sensitivity to paclitaxel in 5-8F cells over-expressing EphA2, which indicated that the PI3K/Akt pathway is involved in EphA2-mediated paclitaxel sensitivity. The current study demonstrated that EphA2 mediates sensitivity to paclitaxel via the regulation of the PI3K/Akt signalling pathway in NPC.

## Introduction

Paclitaxel is used as a first-line chemotherapeutic agent for a broad spectrum of solid malignancies. It exhibits significant anticancer activity. Since the original approval for its clinical application, paclitaxel is now routinely used in the inductive, adjuvant, neoadjuvant and metastatic environment in a number of human cancers including nasopharyngeal carcinoma (NPC) (1–3). Despite its extensive range of applications, the clinical efficiency of paclitaxel is limited by the emergence of resistant cancer cells, which ultimately leads to tumour recurrence and a poor prognosis (4). NPC is a prevalent type of human cancer in Southern China and Southeast Asia, with a clear racial and geographic preponderance (5). Chemotherapy is currently an important therapeutic option for NPC (3,6). However, resistance to chemotherapeutic agents, including paclitaxel is a significant obstacle in the treatment of NPC. Therefore, it is essential to identify molecules associated with paclitaxel resistance and to clarify mechanisms by which they confer resistance in cancer cells, in order to develop novel therapeutic alternatives for use in patients with NPC who have developed paclitaxel resistance.

Ephrin receptors (Ephs) are the largest subfamily of transmembrane receptor tyrosine kinases in the human genome. Eph receptors are classified into Eph type A and B (EphA and EphB) based on sequence homology and binding capacity for two different species of membrane-anchored ephrin ligands (7). EphA2 is a member of EphA receptor tyrosine kinase family. It has a low level of expression in certain noncancerous epithelial cells (8). However, previous studies have demonstrated that the expression of EphA2 is elevated in a large number of human epithelial malignancies, and that elevated EphA2 expression is associated with malignant transformation and a poor prognosis (9–14). Gain- and loss-of-function experiments have shown that abnormal activation of the EphA2 signalling pathway promotes carcinogenesis, indicating that it may act as an oncogene (13,15). EphA2 has been shown to be involved in a number of behaviours associated with malignant cells, including malignant cell transformation, proliferation, angiogenesis, invasion and metastasis (13,16–18).

There is evidence that EphA2 modulates the sensitivity of cancer cells to chemotherapeutic agents. A small number of studies in ovarian and prostate cancer have indicated that EphA2 silencing leads to increased sensitivity to the anticancer drug, paclitaxel (19–21). In addition, given the widespread expression of EphA2 in epithelial carcinoma, novel therapeutic compounds conjugated with the EphA2 receptor may be an effective way to target paclitaxel to cancer cells (21,22). A previous study indicated that EphA2 protein expression is increased in specimens from patients with NPC, and that increased expression of EphA2 is associated with clinical progression of this disease. Furthermore, EphA2 silencing significantly inhibited behaviours associated with malignant transformation and enhanced the sensitivity of NPC cells to paclitaxel *in vitro* (23). Therefore, EphA2 may be a promising molecular target with which to attempt to reverse paclitaxel resistance. However, the molecular mechanisms underlying EphA2-mediated paclitaxel resistance in NPC remain unclear.

## Materials and methods

### Antibodies and reagents

Rabbit EphA2 polyclonal antibodies and mouse β-actin monoclonal antibodies were obtained from Santa Cruz Biotechnology, Inc., (Dallas, TX, USA). Rabbit Akt, phosphor-Akt (p-Akt), p21, cyclin-dependent kinase 2 (CDK2) and Cyclin E monoclonal antibodies, and mouse p27, retinoblastoma protein (Rb), phosphor-Rb, glycogen synthase kinase-3β (GSK-3β) and phosphor-GSK-3β monoclonal antibodies, and the PI3K/Akt signalling pathway small molecule inhibitor, LY294002, were obtained from Cell Signaling Technology, Inc., (Danvers, MA, USA). Paclitaxel was obtained from Bristol-Myers Squibb (New York, NY, USA). The EphA2 cDNA-pEGFP-N1 expression plasmid and pEGFP-N1 vector plasmid were obtained from GeneChem Co., Ltd. (Shanghai, China). Lipofectamine^®^ 2000 Transfection Reagent and Opti-MEM^®^ I Reduced-Serum Medium were obtained from Invitrogen Life Technologies (Carlsbad, CA, USA). 10% foetal bovine serum (FBS) and RPMI-1640 medium were obtained from Hyclone Laboratories, Inc. (Logan, UT, USA). Cell Counting kit-8 (CCK-8), 100 IU/ml penicillin, 100 IU/ml streptomycin, Annexin V-fluorescein isothiocyanate, propidium iodide and BeyoECL Plus Detection system were obtained from Beyotime Institute of Biotechnology (Jiangsu, China). Polyvinylidene fluoride membranes (PVDF) were obtained from EMD Millipore (Billerica, MA, USA).

### Cell lines and culture conditions

5-8F NPC cells were provided by the Cell Center of Central South University, (Changsha, China). 5-8F cells were cultured as a monolayer in RPMI-1640 media with 10% FBS, 100 IU/ml streptomycin and 100 IU/ml penicillin at 37°C in a humidified cell incubator with 5% CO_2_. 5-8F cells in the exponential growth phase were used for subsequent experiments.

### Plasmid construction, transient transfection and efficiency validation

EphA2-specific cDNA lentiviral plasmids (EX-A0125-Lv105, GeneCopoeia, Guangzhou, China) are pools of concentrated, transduction-ready viral particles designed to overexpress EphA2 gene in human NPC 5-8F cells. 5-8F NPC cells (5×10^4^) were seeded in triplicate in 12-well plates and allowed to grow for 24 h. EphA2 cDNA plasmids (2 μg) or empty vectors (2 μg) were transfected into 5-8F NPC cells using Lipofectamine 2000 Transfection Reagent according to the manufacturer’s instructions. At 6 h, the initial transfection medium was changed for fresh medium. The expression of EphA2 in 5-8F cells from each group was assessed using western blotting at 72 h post-infection.

### Western blotting

Western blotting was performed as described previously (9,24). Briefly, equal quantities of total protein samples were separated by 12% sodium dodecyl sulphate-polyacrylamide gel electrophoresis, transferred to a PVDF membrane and incubated with the primary and secondary antibodies. The signalling intensity was visualized using the BeyoECL Plus Detection system. All experiments were performed three times.

### Paclitaxel cytotoxicity assays

Cells (3×10^3^) were separately seeded into 96-well plates in triplicate. At 24 h, cells were treated with varying concentrations of paclitaxel (0, 0.001, 0.01, 0.1, 1, 5, 10, 20 and 30 nM/l) and incubated for a further 48 h. The optical density values of each group were determined by CCK-8 assays. Each experiment was performed in triplicate.

### Assessment of cell cycle and apoptosis by flow cytometry (FCM)

Cells (2×10^5^) from each group were grown in triplicate in 6-well plates for 24 h prior to exposure to 0.1 nM/l paclitaxel for 48 h. Cells were harvested and processed as described previously (24).

### Statistical analysis

Statistical tests were conducted with SPSS 17.0 software (SPSS Inc., Chicago, IL, USA). Quantitative data are presented as the mean ± standard deviation. Differences between groups were compared using a paired t-test. P<0.05 was considered to indicate a statistically significant difference.

## Results

### EphA2 regulates the sensitivity of NPC cells to paclitaxel in vitro

**Figure 1:**
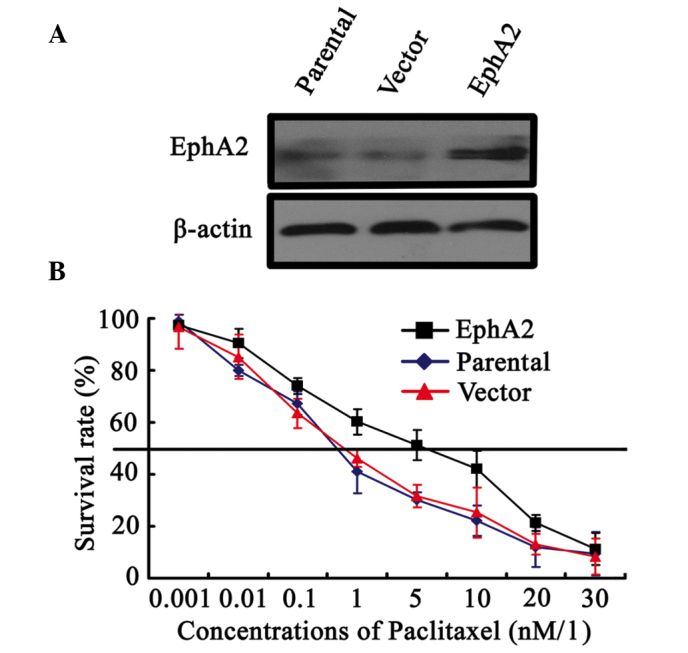
Effects of EphA2 on paclitaxel sensitivity of nasopharyngeal carcinoma 5-8F cells *in vitro*. (A) Western blot analysis was conducted to confirm the efficiency of EphA2 over-expression. (B) Proliferation assay curves of each group. EphA2, ephrin type-A receptor 2.

A preliminary study demonstrated that EphA2 silencing led to increased sensitivity of 5-8F NPC cells to paclitaxel *in vitro* (23). To confirm the association between EphA2 and NPC sensitivity to paclitaxel, an EphA2 cDNA-pEGFP-N1 expression plasmid was used to upregulate EphA2 expression in 5-8F NPC cells. As shown in Fig. 1A, EphA2 was demonstrated to be successfully upregulated in EphA2 cDNA plasmid-transfected 5-8F cells compared with parent and vector plasmid-transfected 5-8F cells. Following paclitaxel stimulation with varying concentrations for 48 h, paclitaxel IC_50_ values in EphA2 cDNA plasmid-transfected, parent and vector plasmid-transfected 5-8F cells were 3.8±0.52, 1.3±0.06 and 1.4±0.05 nM/l, respectively, indicating that EphA2 upregulation enhanced the survival of 5-8F NPC cells compared with control cells exposed to the same concentrations of paclitaxel (Fig. 1B). These results confirmed the involvement of EphA2 in the sensitivity of NPC cells to paclitaxel.

### EphA2 over-expression regulates paclitaxel-mediated cell cycle progression but not apoptosis in NPC

**Figure 2:**
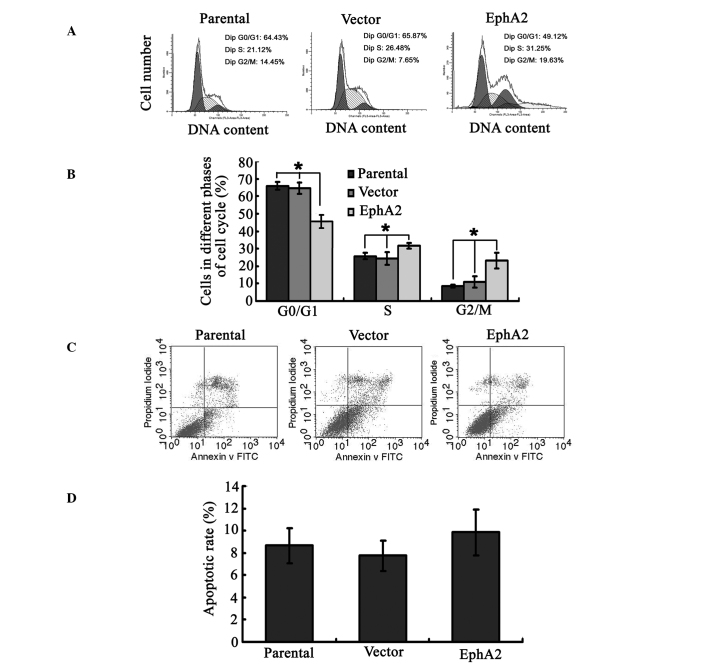
Effect of EphA2 over-expression on cell-cycle distribution and apoptosis following exposure to paclitaxel. (A) Graphs of cell cycle distribution in each group. (B) Percentages of cells in different cell cycle phases in each group. (C) Graphs showing apoptosis in each group. (D) Percentage of apoptotic cells in each group. Results are presented as the mean ± standard deviation of at least three independent experiments. ^*^P<0.05. FITC, fluorescein isothiocyanate; EphA2, ephrin type-A receptor 2.

To investigate the mechanisms underlying EphA2-regulated sensitivity of NPC cells to paclitaxel, changes in cell cycle progression and apoptosis in NPC 5-8F cells, following over-expression of EphA2 and the administration of paclitaxel, were assayed by FCM. The percentage of cells in G0/G1 phase in the 5-8F cells transfected with the EphA2 cDNA plasmid was significantly reduced compared with that in the parent and vector plasmid transfected 5-8F cells (45.76±3.89 compared with 64.52±3.31 and 65.85±2.28%, respectively; P<0.05). By contrast, the percentage of cells in the S phase (31.56±1.59 compared with 24.55±3.64 and 25.76±1.89%, respectively; P<0.05) and the G2/M phase (23.10±4.55 compared with 10.94±3.27 and 8.39±0.81%, respectively; P<0.05) were significantly increased in cells over-expressing EphA2 compared with the other groups (Fig. 2A and B). However, the percentage of apoptotic cells in these three groups was not significantly different, with that of the EphA2-over-expressed group (9.84±2.08) similar to those of the parental (8.66±1.5) and vector (7.74±1.34%) groups (P>0.05) (Fig. 2C and D). These results demonstrate that EphA2 over-expression regulated paclitaxel-mediated cell-cycle progression but not apoptosis in NPC cells.

### EphA2 affects NPC cell-cycle progression via regulation of p21, p27 and p-Rb protein, but not CDK2 and Cyclin E

**Figure 3:**
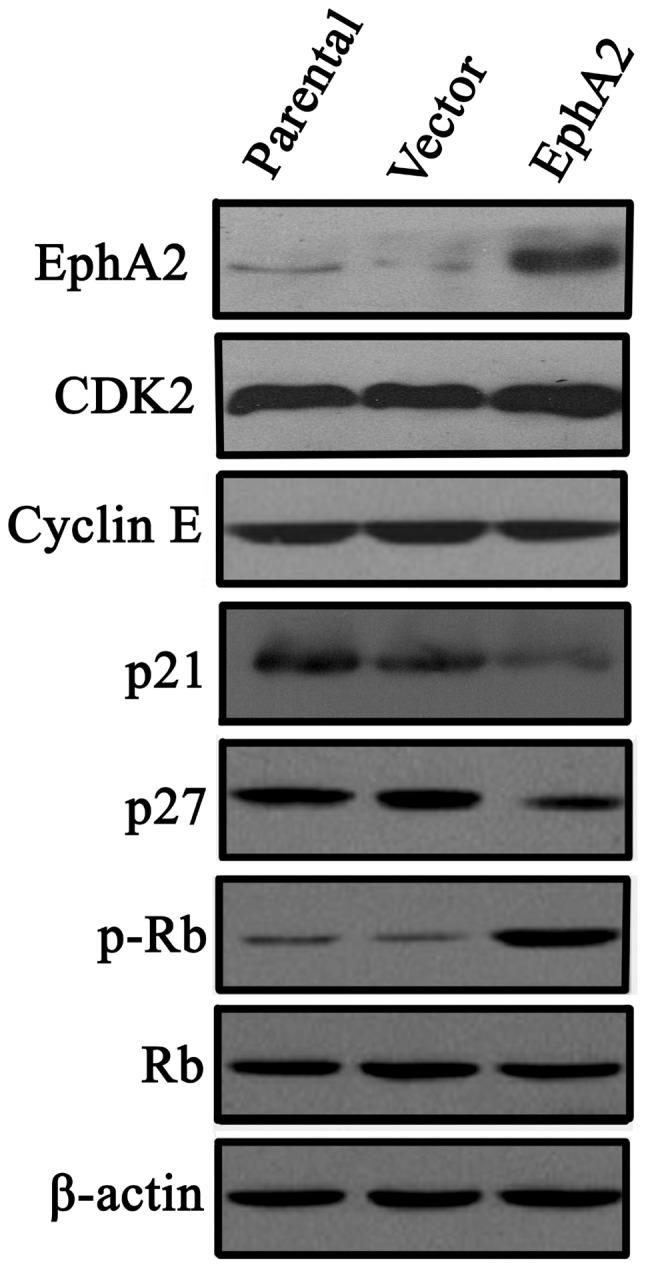
Effect of EphA2 over-expression on cyclin-dependent kinase inhibitors, p21Cip1 and p27Kip1, in NPC 5-8F cells. Western blot analysis was used to detect the expression of p21Cip1, p27Kip1 CDK2, Cyclin E and p-Rb in NPC 5-8F and CNE-2 nasopharyngeal carcinoma cells. All data were obtained by three independent experiments, which produced similar results. EphA2, ephrin type-A receptor 2; CDK, cyclin-dependent kinase; Rb, retinoblastoma protein; p-Rb, phosphorylated Rb; NPC, nasopharyngeal carcinoma.

Since EphA2 expression was associated with changes in cell cycle progression following administration of paclitaxel, the regulation of cell cycle factors by EphA2 was investigated. Western blot analyses indicated that ectopic expression of EphA2 did not influence the expression of the cell cycle promoters, CDK2 and Cyclin E, whereas the expression of cyclin-dependent kinase inhibitors, p21 and p27, were significantly downregulated. In addition, the expression of inactive p-Rb was increased without a change in the total expression of Rb, which is also an inhibitory factor in cell cycle progression (Fig. 3).

### Paclitaxel stimulation and EphA2 over-expression results in activation of the PI3K/Akt signalling pathway in NPC cells

**Figure 4:**
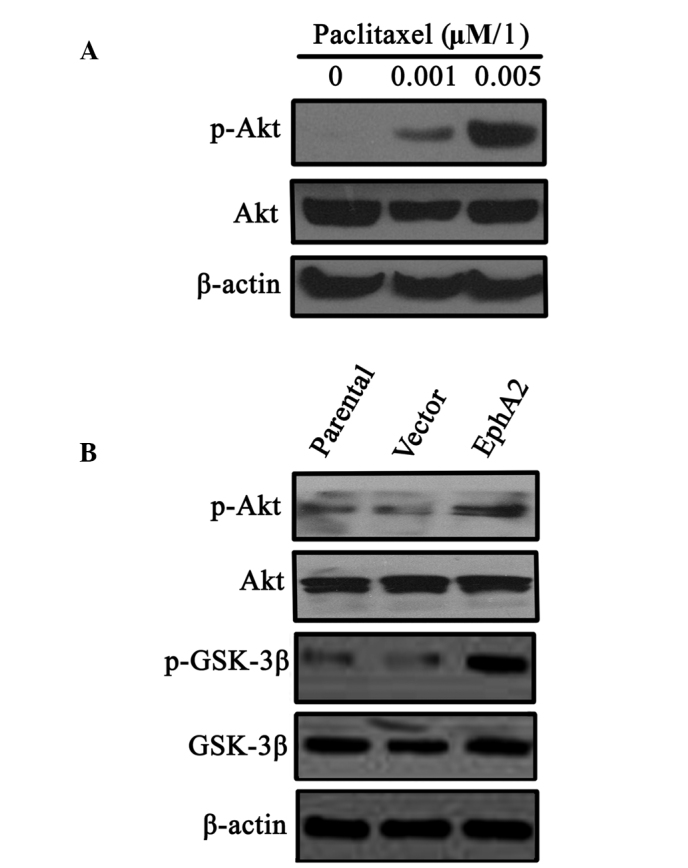
Effect of paclitaxel stimulation and EphA2 overexpression on activation of the PI3K/Akt signalling pathway in NPC 5-8F cells. (A) NPC 5-8F cells were treated with varying concentrations of paclitaxel and PI3K/Akt pathway signalling molecules (total Akt, p-Akt) were measured by western blot analysis. (B) EphA2 over-expression stimulates the PI3K/Akt pathway. The total quantity Akt, p-Akt, GSK-3β and p-GSK-3β was determined by western blot analysis. EphA2, ephrin type-A receptor 2; PI3K, phosphoinositide 3-kinase; p-Akt, phosphorylated Akt; GSK-3β, glycogen synthase kinase-3β; p-GSK-3β, phosphorylated-GSK-3β.

It has been reported that abnormal activation of the PI3K/Akt signalling pathway is involved in sensitivity to paclitaxel in a number of human malignancies. Therefore, the involvement of the PI3K/Akt signalling pathway in EphA2-mediated NPC cell sensitivity to paclitaxel was investigated. The results demonstrated that continuous paclitaxel stimulation induced an increase in p-Akt expression in 5-8F NPC cells (Fig. 4A). Furthermore, over-expression of EphA2 in 5-8F cells also increased p-Akt expression and the expression of its downstream signalling molecule, p-GSK-3β, indicating that there was aberrant activation of the PI3K/Akt signalling pathway (Fig. 4B). These results suggest that the PI3K/Akt pathway may be involved in the regulation of EphA2-mediated NPC sensitivity to paclitaxel.

### PI3K/Akt signalling pathway is involved in EphA-mediated sensitivity to paclitaxel

**Figure 5:**
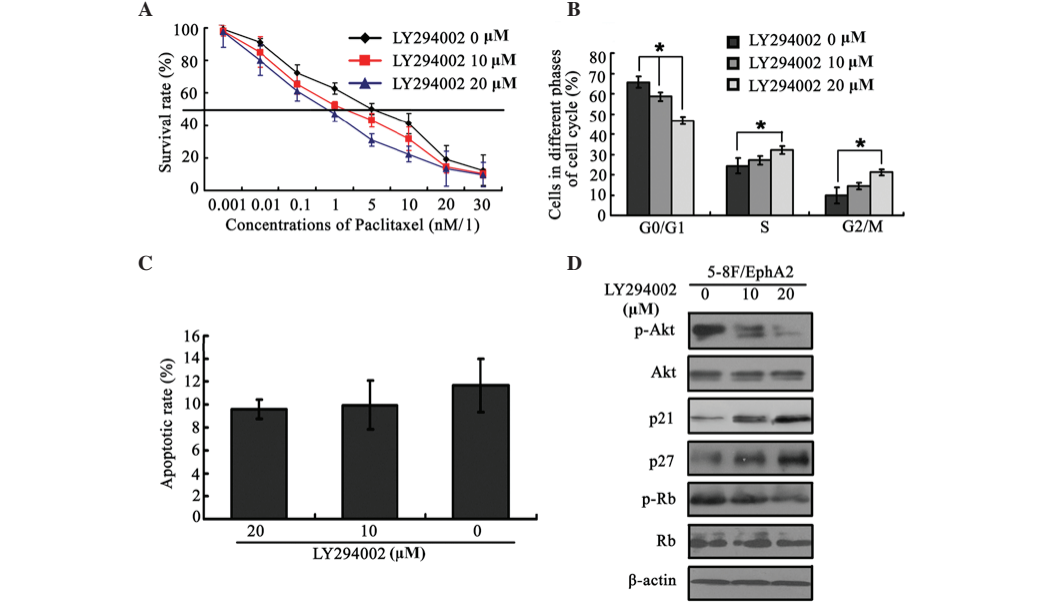
EphA2 mediated paclitaxel sensitivity in NPC 5–8 cells via modulation of the PI3K/Akt signalling pathway. (A) PI3K/Akt signalling pathway inhibitor, LY294002, reverses paclitaxel resistance caused by EphA2 over-expression. (B) Effect of LY294002 on the cell-cycle distribution in EphA2 over-expressing NPC cells pre-treated with paclitaxel. (C) Effect of LY294002 on the apoptotic rate in NPC cells over-expressing EphA2, pretreated with paclitaxel. (D) LY294002 restores the changes in expression of cyclin-dependent kinase inhibitors, p21Cip1 and p27Kip1, caused by EphA2 over-expression. ^*^P<0.05. EphA2, ephrin type-A receptor 2; NPC, nasopharyngeal carcinoma; PI3K, phosphoinositide 3-kinase; p-Akt, phsophorylated Akt; Rb, retinoblastoma protein; p-Rb, phosphorylated Rb.

To further investigate the role of the PI3K/Akt signalling pathway in EphA2-mediated sensitivity to paclitaxel, a small molecule inhibitor of the PI3K/Akt signalling pathway, LY294002, was used to block this pathway in 5-8F cells over-expressing EphA2, and to observe whether EphA2-mediated changes in sensitivity to paclitaxel are reversed in EphA2-over-expressing 5-8F cells. As shown in Fig. 5A, LY294002 significantly restored sensitivity to paclitaxel, which had been reduced by EphA2 over-expression, in a dose-dependent manner. This was accompanied by corresponding changes in the cell-cycle phase distribution (Fig. 5B) but was not associated with changes in the percentage of apoptotic rate (Fig. 5C). Furthermore, the changes in expression of cell-cycle regulatory factors p21, p27 and p-Rb resulting from EphA2 over-expression were also reversed by the addition of LY294002 (Fig. 5D). These results provide further evidence that the PI3K/Akt pathway is involved in EphA2-mediated sensitivity to paclitaxel.

## Discussion

The development of paclitaxel resistance is a characteristic feature in NPC progression and is associated with a poor prognosis and increased mortality in patients with advanced NPC. Therefore, it is important to identify molecules and their downstream signalling pathways that permit cancer cells to evade the cytotoxic effects of paclitaxel and to maintain unregulated growth.

Existing evidence has demonstrated that EphA2 expression is associated with cancer cell metastasis. Numerous reports have focused on the impact of EphA2 on this process (25), and a previous study also showed that EphA2 knockdown inhibited metastasis in NPC *in vitro* (23). In the present study, EphA2 was found to modulate the sensitivity of NPC cells to paclitaxel, which was consistent with previous studies that have reported that EphA2 inhibition leads to increased paclitaxel sensitivity in ovarian cancer (19,26). Recently, a novel treatment, in which an agent targeted against EphA2 was conjugated with paclitaxel (22). It demonstrated significantly enhanced antitumour efficacy compared with paclitaxel alone in a xenograft animal model of prostate cancer (21). However, a literature review demonstrated that there has been little investigation into the association between abnormal expression of EphA2 and paclitaxel resistance. Paclitaxel exerts its effect partly by inducing cell cycle arrest and activating proapoptotic signalling pathways (27). The results from the current study showed that EphA2 over-expression led to downregulation of the cyclin-dependent kinase inhibitors, p21 and p27, and an increase in the expression of the inactive p-Rb. These changes expedited NPC cell cycle progression but did not affect apoptosis. Thus, EphA2 renders NPC cells resistant to paclitaxel by affecting NPC cell-cycle progression. These results indicate that EphA2 is involved in the modulation of sensitivity to paclitaxel in human malignancies, including NPC.

Investigation of the mechanisms underlying this effect showed that the PI3K/Akt signalling pathway is involved in EphA2-mediated sensitivity to paclitaxel of NPC. A number of groups have reported that activation of PI3K/Akt may protect cancer cells against the cytotoxic effects of anticancer drugs, including paclitaxel (28–31). Abnormal activation of PI3K/Akt during chemotherapy is one of the primary causes for the development of chemotherapeutic resistance. Therefore, PI3K/Akt activity following paclitaxel stimulation and EphA2 upregulation was investigated. It was shown that PI3K/Akt was activated by paclitaxel stimulation and ectopic expression of EphA2 in 5-8F NPC cells. PI3K/Akt is known to regulate the expression of certain mediators of cell cycle progression, and their expression in numerous human cancers has been shown to be associated with cancer cell survival, chemoresistance and radioresistance (32). In the present study, it was demonstrated that ectopic expression of EphA2 in combination with administration of paclitaxel enhanced NPC cell cycle progression via downregulation of the cell cycle regulators, p21 and p27, and upregulation of the inactive p-Rb. This conferred survival advantages to 5-8F NPC cells, leading to paclitaxel resistance. However, the PI3K/Akt inhibitor, LY294002, reversed the paclitaxel resistance, along with changes in mediators of cell cycle progression, which were caused by ectopic expression of EphA2. Therefore, EphA2 inhibition is a candidate for combination treatment with the anticancer agent, paclitaxel, which induces activation of the PI3K/Akt signalling pathway. A previous study demonstrated that EphA2 regulates the growth of cancer cells via numerous signalling pathways, reflecting its complicated regulatory network (33). The present results suggest that abnormal activation of the PI3K/Akt pathway caused by EphA2 over-expression is part of the mechanism underlying the EphA2-mediated paclitaxel resistance in NPC cells. Another study into the molecular mechanisms underlying paclitaxel resistance showed that dysfunction of multidrug resistance (MDR)-1 gene and its encoding protein P-glycoprotein, leads to the efflux of paclitaxel, thus disrupting paclitaxel retention (34). A study by our group (Yunyun Wang *et al*, unpublished data) showed that EphA2 regulates the chemoresistance of paclitaxel by mediating the expression of MDR-1. Thus, combination therapy consisting of EphA2 targeted knockdown and paclitaxel has synergistic effects and may represent a promising therapeutic strategy for patients with advanced NPC.

In conclusion, the present study demonstrated the efficacy of EphA2 inhibition on enhancement of chemosensitivity to paclitaxel in NPC *in vitro*. Over-expression of EphA2 led to increased PI3K/Akt activity, resulting in promotion of cell cycle progression in NPC cells, whilst inhibition of PI3K/Akt reversed the EphA2-mediated reduction in paclitaxel sensitivity. Although additional *in vivo* studies and clinical trials are required to explore the efficacy and safety, the cytotoxic effect of EphA2 inhibition in combination with paclitaxel may provide a novel treatment strategy for patients with advanced NPC.
